# Association Between Angiotensin‐Converting Enzyme (ACE) Gene Insertion/Deletion (I/D) Polymorphism Genotypes With Brain Volume and Hypertension in Alzheimer's Disease—A Retrospective Study

**DOI:** 10.1002/kjm2.70046

**Published:** 2025-05-15

**Authors:** Bin‐Tse Lin, Ching‐Fang Chien, Ling‐Chun Huang, Yuan‐Han Yang

**Affiliations:** ^1^ Neuroscience Research Center, Kaohsiung Medical University Kaohsiung Taiwan; ^2^ Department of Post‐Baccalaureate Medicine Kaohsiung Medical University College of Medicine Kaohsiung Taiwan; ^3^ Department of Neurology Kaohsiung Medical University Hospital Kaohsiung Taiwan; ^4^ Department of Neurology Kaohsiung Medical University Gangshan Hospital Kaohsiung Taiwan

**Keywords:** Alzheimer's disease, angiotensin‐converting enzyme, brain volume, hypertension

## Abstract

This study investigates the role of the *ACE* I/D polymorphism in Alzheimer's disease (AD) patients, particularly in relation to hypertension and its influence on brain volume. Seventy‐seven AD patients, diagnosed based on Aging and Alzheimer's Association criteria, were enrolled from the Kaohsiung Municipal Ta‐Tung Hospital Dementia Cohort. *ACE* I/D genotypes were identified through polymerase chain reaction, and various factors such as age, sex, education, brain volume, and neuropsychological test scores were analyzed. The results indicated that *ACE* genotypes, presence of apolipoprotein epsilon 4 (*APOEε4*), and brain volume did not significantly differ between patients with and without hypertension. While age and sex were associated with gray matter volume, cerebrospinal fluid volume correlated with age, sex, and hypertension. Total cranial volume was linked to sex, and the cerebrospinal fluid‐to‐total intracranial volume ratio was influenced by sex and education. Overall, *ACE* I/D genotypes and *APOEε4* did not have a significant impact on brain volume in AD patients, regardless of hypertension status. Instead, brain atrophy was associated with sex, age, education, and hypertension. These findings suggest that although *ACE* may not significantly influence brain volume in AD patients, further large‐scale studies are needed to clarify its role in AD pathogenesis.

## 1 Introduction

Alzheimer's disease (AD) is a multifactorial disorder influenced by genetic factors. The angiotensin‐converting enzyme (*ACE*) gene regulates blood pressure by converting angiotensin I to angiotensin II, which stimulates aldosterone to maintain fluid and electrolyte balance. Beyond cardiovascular disease, ACE has been implicated in AD through its role in amyloid‐beta (Aβ) clearance [1, 2]. Toxic Aβ aggregation disrupts neuronal function, contributing to cognitive decline. Notably, ACE inhibitors, commonly used for hypertension, may elevate cerebral Aβ levels. A previous study in African American patients linked elevated blood angiotensin II to reduced hippocampal and cortical volumes, structures critical to cognitive function in early AD [3]. However, the precise role of ACE in AD pathogenesis remains unclear.

A key genetic feature is the *ACE* insertion/deletion (I/D) polymorphism in intron 16 [4], which influences both hypertension and AD risk, with population‐specific effects. The D allele is linked to hypertension in northeastern China [5] and Japan [6], though no such association was found in Italy [7]. In AD, the I/D genotype increased susceptibility in a Kashmiri population [8], whereas the I/I genotype was associated with a lower AD risk in Taiwan [9]. Functionally, the D/D genotype elevates angiotensin IV levels in the neocortex and hippocampus, potentially enhancing memory [10, 11], while the I allele is linked to increased AD risk and neuropsychiatric symptoms [12]. Despite the D/D genotype being associated with higher ACE protein levels, the I allele, which contains an Alu sequence in intron 16, enhances transcription activity by ~70% [9, 13].

The *ACE* gene's impact on brain volume and AD remains underexplored. The I allele correlates with reduced age‐related white matter (WM) changes, particularly in Taiwanese females [14], whereas D allele carriers with mild cognitive impairment exhibit lower fractional anisotropy in key brain regions, suggesting WM damage [15]. Given the complex relationship between *ACE* polymorphisms and brain structure, this study examines the influence of the *ACE* I/D polymorphism on brain volume in AD patients with and without hypertension.

## 2 Methods

### 2.1 Study Population

This retrospective chart review included 77 AD patients (aged 59–96) from the Kaohsiung Municipal Ta‐Tung Hospital Dementia Cohort. AD diagnosis followed the National Institute of Neurological and Communicative Disorders and Stroke–Alzheimer's Disease and Related Disorders Association criteria [16] and was supported by neuropsychological tests, including the Mini‐Mental State Examination [17], Cognitive Assessment Screening Instrument [18], Neuropsychiatric Inventory [19], and Clinical Dementia Rating scale [20]. Patients with mixed‐type dementia or other cognitive‐impairing conditions were excluded. Hypertension was defined as diastolic blood pressure ≥ 90 mmHg, systolic blood pressure ≥ 140 mmHg, or current antihypertensive use.

### 2.2 Brain Volume Measurement

MRI images were analyzed using Statistical Parametric Mapping 12 (SPM12) in MATLAB [21]. Gray matter (GM), WM, and cerebrospinal fluid (CSF) were segmented using tissue probability maps, and total volumes were computed by multiplying voxel intensity by tissue probability, adjusted for voxel size (mm^3^). GM and WM volumes were normalized by total intracranial volume (TIV) to account for individual brain size variations, ensuring accurate intergroup comparisons [22].

### 2.3 ACE Genotyping

*ACE* wildtype and I/D genotypes were identified using PubMed gene database sequences, aligned with patient samples. Pyrosequencing (Pyrosequencer) was performed on 10 ng DNA amplified via PCR (target fragment: 250 bp) [23].

### 2.4 APOE Genotyping

*APOE* genotypes were determined by analyzing SNPs rs429358 and rs7412 using Axiom Analysis Suite and PLINK software [24].

### 2.5 Statistical Analysis

SPSS version 20.0 (IBM, Armonk, NY, USA) was used for statistical analysis. Categorical data were reported as counts (%), and continuous data as mean ± standard deviation. Chi‐square tests analyzed categorical variables. Logistic regression assessed the relationship between brain volume and *ACE* genotype, adjusting for age, sex, education, *APOEε4* status, and hypertension. Statistical significance was set at *p* < 0.05.

## 3 Results

A total of 77 AD patients were analyzed, with 34 (44.2%) having the I/D genotype, 35 (45.5%) the I/I genotype, and 8 (10.4%) the D/D genotype. Table 1 presents the demographic data, comorbidities, neuropsychological test results, and brain volume by *ACE* genotype. No significant differences were observed among the *ACE* I/D polymorphism groups regarding age, sex, education, comorbidities (diabetes, hypertension, hyperlipidemia, stroke), neuropsychological performance, or brain volume.

**TABLE 1:** Demographic data, comorbidities, neuropsychological tests, and brain volume of the patients by *ACE* genotype.

*ACE* genotype	Total	I/D	I/I	D/D	*p*
*N* [*n* (%)]	77	34 (44.2)	35 (45.5)	8 (10.4)	0.952
Age (years) [mean (SD)]	78.55 (7.0)	78.4 (7.6)	78.7 (7.0)	78.5 (4.4)	0.974
Sex (female) [*n* (%)]	48 (62.3)	25 (52.1)	18 (37.5)	5 (10.4)	0.166
Education (years) [mean (SD)]	7.6 (5.3)	6.9 (5.3)	7.8 (5.4)	9.4 (5.6)	0.516
Diabetes mellitus [*n* (%)]	20 (26.0)	7 (35.0)	11 (55.0)	2 (10.0)	0.589
Hypertension [*n* (%)]	29 (37.7)	11 (47.8)	10 (43.5)	2 (8.7)	0.896
Hyperlipidemia [*n* (%)]	28 (36.4)	15 (53.6)	9 (32.1)	4 (14.3)	0.198
Stroke [*n* (%)]	26 (33.8)	11 (42.3%)	13 (50.0)	2 (7.7%)	0.785
MMSE [mean (SD)]	15.6 (6.5)	15.6 (6.2)	15.4 (6.7)	16.8 (7.1)	0.865
CASI [mean (SD)]	56.0 (20.3)	59.9 (19.0)	53.8 (21.0)	61.5 (23.8)	0.594
NPI [mean] (SD)]	14.7 (16.0)	12.8 (13.8)	19.6 (20.1)	9.6 (9.4)	0.204
CDRSB [mean (SD)]	6.32 (3.5)	6.4 (3.6)	6.5 (3.5)	5.3 (3.1)	0.690
CDR [*n* (%)]					0.958
0.5	23 (29.8)	10 (43.5)	10 (43.5)	3 (13.0)	
1	38 (49.4)	16 (42.1)	18 (47.4)	4 (10.5)	
2	16 (20.8)	8 (50.0)	7 (43.8)	1 (6.3)	
Gray matter (GM) [mean (SD)]	0.383 (0.096)	0.384 (0.093)	0.394 (0.100)	0.330 (0.090)	0.234
White matter (WM) [mean (SD)]	0.504 (0.084)	0.504 (0.063)	0.502 (0.106)	0.518 (0.050)	0.888
Cerebrospinal fluid (CSF) [mean (SD)]	0.481 (0.102)	0.463 (0.119)	0.499 (0.089)	0.484 (0.071)	0.351
Total intracranial volume (TIV) [mean (SD)]	1.368 (0.127)	1.350 (0.139)	1.394 (0.122)	1.331 (0.074)	0.250
GM/TIV [mean (SD)]	0.280 (0.067)	0.274 (0.073)	0.284 (0.062)	0.290 (0.065)	0.744
WM/TIV [mean (SD)]	0.370 (0.059)	0.366 (0.067)	0.373 (0.052)	0.374 (0.055)	0.861
CSF/TIV [mean (SD)]	0.350 (0.059)	0.361 (0.050)	0.343 (0.068)	0.336 (0.046)	0.359

Abbreviations: *ACE*, angiotensin‐converting enzyme; CASI, cognitive abilities screening instrument; CDR‐SB, Clinical Dementia Rating‐Sum of Boxes; MMSE, mini‐mental state examination.

Table 2 compares *ACE* I/D genotypes, *APOEε4* status, and brain volumes between AD patients with and without hypertension. No significant differences were found between these groups.

**TABLE 2:** Brain volume measurements, *ACE* I/D genotypes, and *APOEε4* status of the patients with or without hypertension.

	AD with hypertension	AD without hypertension	*p*
*N* [*n* (%)]	23 (29.9)	54 (70.1)	
*ACE* I/D, [*n* (%)]	11 (14.3)	23 (29.9)	0.677
*ACE* I/I [*n* (%)]	10 (13.0)	25 (32.5)	0.823
*ACE* D/D [*n* (%)]	2 (2.6)	6 (7.8)	0.754
*APOEε4*(+) [*n* (%)]	8 (10.4)	16 (20.8)	0.660
*APOEε4*(−) [*n* (%)]	15 (19.5)	38 (49.4)	0.660
Gray matter (GM) [mean (SD)]	0.372 (0.096)	0.387 (0.097)	0.546
White matter (WM) [mean (SD)]	0.512 (0.072)	0.501 (0.089)	0.579
Cerebrospinal fluid (CSF) [mean (SD)]	0.508 (0.106)	0.470 (0.100)	0.142
Total intracranial volume (TIV) [mean (SD)]	1.392 (0.144)	1.358 (0.119)	0.277
GM/TIV [mean (SD)]	0.277 (0.069)	0.282 (0.067)	0.796
WM/TIV [mean (SD)]	0.372 (0.072)	0.369 (0.053)	0.800
CSF/TIV [mean (SD)]	0.351 (0.058)	0.350 (0.059)	0.967

Abbreviations: *ACE*, angiotensin‐converting enzyme; *APOEε4*, apolipoprotein epsilon 4.

Logistic regression analysis (Table 3) adjusted for age, sex, education, *ACE* I/D genotypes, *APOEε4* status, and hypertension, revealing that age (*p* < 0.001) and sex (*p* = 0.014) were significantly associated with GM volume; age (*p* < 0.001), sex (*p* < 0.001), and hypertension (*p* = 0.033) with CSF volume; and sex with TIV (*p* < 0.001). Additionally, sex (*p* = 0.023) and education (*p* = 0.029) were significantly associated with CSF normalized to TIV. No significant associations were found between *ACE* I/D genotypes and brain volumes after adjustment.

**TABLE 3:** Logistic regression analyses of brain volume referring to *ACE* I/D genotype.

Brain volume	Gray matter (GM)		White matter (WM)		Cerebral spinal fluid (CSF)		Total intracranial volume (TIV)		GM/TIV		WM/TIV		CSF/TIV	
	*β*	*p*	*β*	*p*	*β*	*p*	*β*	*p*	*β*	*p*	*β*	*p*	*β*	*p*
Age	−0.406	< 0.001*	−0.009	0.941	0.365	< 0.001*	−0.016	0.845	0.186	0.132	−0.199	0.104	−0.009	0.937
Sex	0.281	0.014*	0.070	0.593	0.554	< 0.001*	0.712	< 0.001*	0.130	0.324	0.141	0.278	−0.292	0.023*
Education	0.032	0.780	0.062	0.642	0.010	0.925	0.074	0.410	−0.108	0.418	−0.156	0.237	0.283	0.029*
*APOEε4*	0.011	0.918	0.145	0.234	−0.109	0.247	0.017	0.833	0.013	0.914	−0.078	0.511	0.065	0.573
*ACE*	−0.109	0.296	−0.011	0.931	−0.005	0.956	−0.094	0.255	0.038	0.752	0.062	0.608	−0.107	0.361
Hypertension	−0.069	0.510	0.070	0.574	0.208	0.033*	0.164	0.051	0.027	0.824	0.081	0.506	−0.114	0.336

Abbreviations: *ACE*, angiotensin‐converting enzyme; *APOEε4*, apolipoprotein epsilon 4.

* *p* < 0.05.

## 4 Discussion

### 4.1 Influence of Age, Sex, Education, and Hypertension

Age and sex significantly affected GM volume, while age, sex, and hypertension influenced cerebrospinal fluid (CSF) volume. TIV was sex‐dependent, and the CSF/TIV ratio was influenced by sex and education. These findings align with prior research linking age‐related cognitive decline, sex differences in dementia prevalence, and lower education levels with heightened AD risk [25, 26, 27].

Hypertension, a key contributor to AD pathology, damages cerebral microvasculature, leading to atrophy and white matter lesions [28]. It also increases stroke risk, which accounts for up to 55% of AD cases linked to hypertension [29]. Antihypertensive treatment can reduce AD risk by 36%–42% [30], reinforcing its role in disease prevention.

### 4.2 Comparison With Previous ACE Polymorphism Studies

Contrary to earlier studies, we found no significant association between *ACE* polymorphisms and brain volume in AD patients. Differences in study design, sample size, and population demographics likely explain these discrepancies. While previous case–control studies used postmortem data from Caucasian or Tunisian cohorts [31, 32], our study focused on living Taiwanese AD patients. Moreover, earlier studies reported that the D/D genotype was linked to increased brain atrophy and cognitive decline, whereas the I/I genotype was associated with larger hippocampal and amygdala volumes. Our findings provide unique insights from an Asian cohort, contributing to the broader understanding of *ACE* polymorphisms in AD.

### 4.3 Interplay Between ACE and APOEε4 Alleles

The interaction between *ACE* and *APOEε4* in AD remains unclear but may involve vascular function and Aβ metabolism. ACE influences blood pressure, increasing cerebrovascular damage risk, while APOEε4 is similarly linked to vascular dysfunction [33]. Additionally, ACE plays a role in Aβ clearance [2], and APOEε4 affects both Aβ deposition and clearance [34], potentially exacerbating AD pathology. Further studies with larger samples are needed to clarify these gene–gene interactions in brain volume changes.

### 4.4 Strengths and Limitations

This study integrates genetic, cardiovascular, and demographic factors to provide a comprehensive analysis of AD‐related brain volume changes. By evaluating multiple variables, including neuropsychological test scores and MRI‐based measurements, we highlight modifiable risk factors, particularly hypertension, for potential intervention.

However, the relatively small sample size may limit generalizability and statistical power. Differences in population characteristics may also explain inconsistencies with prior ACE and APOE findings. Future large‐scale studies are essential to unravel the complex interplay between genetic and non‐genetic factors in AD.

## 5 Conclusion

Our study found no significant association between *ACE* I/D polymorphisms or *APOEε4* status and brain volume in AD patients, even with hypertension. Instead, age, sex, education, and hypertension were key determinants of brain volume, highlighting the interaction of genetic and non‐genetic factors in AD. These results emphasize the role of demographic and cardiovascular factors, particularly hypertension, in brain atrophy. While ACE and APOEε4 did not directly influence brain volume, modifiable risk factors like hypertension may play a crucial role in AD progression. Further research with larger cohorts is needed to better understand the complex influences on AD pathology.

## Conflicts of Interest

The authors declare no conflicts of interest.

## Data Availability

The data supporting the findings of this study are available within the article.
